# Automated tests of ANA immunofluorescence as throughput autoantibody detection technology: strengths and limitations

**DOI:** 10.1186/1741-7015-12-38

**Published:** 2014-03-03

**Authors:** Pier Luigi Meroni, Nicola Bizzaro, Ilaria Cavazzana, Maria Orietta Borghi, Angela Tincani

**Affiliations:** 1 Department of Clinical Sciences & Community Health, University of Milan, Milan, Italy; 2 Istituto Auxologico Italiano, Milan, Italy; 3 Laboratory of Clinical Pathology, Ospedale San Antonio, Tolmezzo, Italy; 4 Rheumatology Unit and Chair, Spedali Civili di Brescia, Università degli Studi di Brescia, Brescia, Italy; 5 Division of Rheumatology, Istituto G. Pini, Piazza C. Ferrari, 1, 20122 Milan, Italy

**Keywords:** Anti-nuclear antibodies, Indirect immunofluorescence, Autoimmunity

## Abstract

Anti-nuclear antibody (ANA) assay is a screening test used for almost all autoimmune rheumatic diseases, and in a number of these cases, it is a diagnostic/classification parameter. In addition, ANA is also a useful test for additional autoimmune disorders. The indirect immunofluorescence technique on monolayers of cultured epithelial cells is the current recommended method because it has higher sensitivity than solid phase assays. However, the technique is time-consuming and requires skilled operators. Automated ANA reading systems have recently been developed, which offer the advantage of faster and much easier performance as well as better harmonization in the interpretation of the results. Preliminary validation studies of these systems have given promising results in terms of analytical specificity and reproducibility. However, these techniques require further validation in clinical studies and need improvement in their recognition of mixed or less common staining patterns.

## Background

Anti-nuclear antibody assay (ANA) is the screening test of choice for diagnosis of almost all systemic autoimmune rheumatic diseases (SARDs) because of its greater sensitivity compared with other assays, even though its specificity is much lower (Box 1) [1]. The gold standard method for ANA detection is still indirect immunofluorescence (IIF) on human epithelial (HEp-2) cells, as the alternative tests cannot display comparable sensitivity [2]. However, the technique is time-consuming and requires skilled operators. This fact together with the widespread increase in ANA requests and the reduction of laboratory facilities because of the budget constriction generated a strong need for advanced automated platforms as in other branches of the laboratory medicine.

### ANA automated reading systems

Currently, at least six commercial systems for the automated reading of ANA IIF are available: Aklides (Medipan, Dahlewitz, Germany), EUROPattern (Euroimmun AG, Luebeck, Germany), Helios (Aesku Diagnostics, Wendelsheim, Germany), Image Navigator (ImmunoConcepts, Sacramento, CA), NOVA View (Inova Diagnostics, San Diego, CA), and Zenit G-Sight (A. Menarini Diagnostics, Florence, Italy).

These systems are based on a composition of different hardware modules combined with mathematical pattern-recognition software algorithms, enabling fully automated image acquisition, analysis, and evaluation of IIF ANA tests.

Samples can be classified as positive or negative and the main IIF pattern recognized (Table 1). In addition, quantitative fluorescence intensity value (equivalent to the end-point titer) can be obtained. To date, 13 studies have been published assessing the reliability of automated IIF analysis as a standardized alternative for the conventional manual visual approach (Table 2) [3-14].

**Table 1:** Types of indirect immunofluorescence pattern identified by the currently available automated systems for anti-nuclear antibody assay

**System**	**Pattern**
Aklides	Homogeneous, speckled, nucleolar, centromeric, nuclear dots, cytoplasmic
EuroPattern	Homogeneous, speckled, nucleolar, centromeric, nuclear dots, cytoplasmic
Helios	Visual recognition by the operator
Image Navigator	Visual recognition by the operator
Nova View	Homogeneous, speckled, nucleolar, centromeric, nuclear dots, cytoplasmic
Zenit G-Sight	Homogeneous, speckled, nucleolar, centromeric, nuclear dots, mitochondrial

**Table 2:** Automated/manual positive–negative agreement (PNA) for each anti-nuclear antibody indirect immunofluorescence reading system, based on 13 published studies

**System**	**Studies, n**	**Patients, n**	**PNA, mean**
Aklides	3	1801	0.95
EuroPattern	2	467	0.97
Helios	1	1005	0.98
Image Navigator	1	3185	0.99
Nova View	2	842	0.95
Zenit G-Sight	3	830	0.92
All systems	1	149	0.96
Total	13	8279	0.97

The reported advantages of these systems include reduction in intra-laboratory and inter-laboratory variability, improvement in correlation between staining patterns with corresponding autoantibody reactivities, higher throughput in laboratory workflows, no requirement for a darkroom, integrated file storage, and easy retrieval of scanned wells.

### Comparison of the available ANA automated reading systems

Although comparable performance between automated and conventional ANA IIF analysis for the interpretation of negative and positive samples has been reported, discrepancies between patterns have been found, especially when systems are able to detect basic patterns only, or when mixed fluorescent patterns are present in the samples [3-14].

Some automated IIF systems present misinterpretation difficulties when antibodies react with a limited and specific cell component, such as Golgi apparatus, nuclear dots, or nuclear membrane [3-14]. Such misinterpretation may have implications in clinical settings, emphasizing the need and importance of visual validation (Table 3).

**Table 3:** **Indirect immunofluorescence patterns detected on HEp-2 cells, with, related antigens and diagnosis**^**a**^

	**Related antigens**	**Related diagnosis**
Nuclear patterns		
Homogeneous	DNA, histones, chromatin/nucleosomes	SLE, drug-induced SLE, JIA
Peripheral/rim or nuclear envelope	Lamins, LAP1/2 gp210, nucleoporin p62; Tpr nuclear envelope and nuclear pore complex antigens	SLE, RA, PBC, myositis, autoimmune liver disease, PAPS
Coarse speckled	U1-snRNP, U2-6 snRNP (Sm), nuclear matrix	MCTD, SLE, Raynaud, SSc, SS, UCTD
Fine speckled	SSA/Ro, SSB/La, common to many antigens	SLE, SS, SSc, myositis, MCTD
Dense fine speckled	DFS70/LEDGF-P75	Healthy subjects and other inflammatory conditions
PCNA	Auxiliary protein proliferating cell nuclear antigen: elongation factor of DNA polymerase δ	SLE, lymphoproliferative diseases, SS
Diffuse speckled with “cloudy” mitoses	Topoisomerase-I	SSc
Centromere	Kinetochore: CENP-A, CENP-B, CENP-C, CENP-F	SSc (limited)
Nucleolar homogeneous	PM/Scl, RNA polymerase, To/Th , B23 phosphoprotein/numatrin	SSc, myositis, overlap myositis/SSc
Nucleolar speckled	RNA polymerase (I to III)	SSc
Nucleolar clumpy	U3-RNP (fibrillarin)	SSc
Multiple/few nuclear dots	Sp100/140, PML bodies, NDP53, p80-coilin, PML bodies	PBC, CAH, SS
Centrosome/centriole (formerly spindle apparatus)	Enolase, ninein, pericentrin	SSc, Raynaud’s phenomenon, inflammatory disease
MSA	NuMA/centrophilin Hseg5	RA, inflammatory conditions; pneumonia (mycoplasma)
Cytoplasmic patterns		
Diffuse homogeneous (nucleoli positive)	Ribosomal proteins	SLE
Fine speckled	Jo-1, SRP, PDH (mitochondria)	Myositides, DM, PBC, interstitial lung disease
Discrete speckled	Endosome (early endosome antigen 1), GW/P bodies, multivesicular bodies/lysosomes	Neurological conditions, SS, SLE, RA, PBC
Golgi complex	Golgi proteins	SLE, SS, RA, overlap syndromes, cerebellar ataxia
Cytoplasmic fibers	Actin, cytokeratin, tropomyosin, vimentin	CAH, DM, infections and other inflammatory diseases

CAH, chronic autoimmune hepatitis; CENP, centromere protein; DM, dermatomyositis; DFS70/LEDGF, dense fine speckled/lens epithelium-derived growth factor; JIA, juvenile idiopathic arthritis; MCTD, mixed connective tissue disease; MSA, mitotic spindle apparatus; PAPS, primary antiphospholipid syndrome; PBC, primary biliary cirrhosis; PCNA, proliferating cell nuclear antigen; PDH, phosphate dehydrogenase; PM, polymyositis; RA, rheumatoid arthritis; Scl, scleroderma; SLE, systemic lupus erythematosus; snRNP, small nuclear ribonuclear protein; SRP, signal recognition particle; SSc, systemic sclerosis; SS, Sjögren’s syndrome; UCTD, undifferentiated connective tissue disease.

^a^Modified from Agmon-Levin *et al*. [15].

Such IIF assays have identifed more than 50 autoantibodies against 30 different nuclear and cytoplasmic antigens [16]. The use of large cultured cells with high rates of mitosis enables adequate pattern recognition by evaluation of the fluorescence distribution during different phases of the cell cycle. In fact, identification of cell cycle dynamics (for example, interphase, mitosis) is crucial both for defining different patterns (such as the fine or large speckled patterns within a speckled staining pattern, the centromere patterns and the PCNA patterns) and for distinguishing between different patterns (for example anti-nuclear membrane from the homogeneous pattern).

Correct identification of different IIF patterns is sometimes diagnostic (for example, the centromere pattern and the PCNA pattern) or may suggest the occurrence of autoantibodies to specific antigens (Table 3). Many sera contain more than one antibody; in such cases, accurate analysis of the different patterns often requires direct evaluation of the slides to enable exact definition of the autoantibody profile in a given patient.

Systemic sclerosis (SSc) represents a paradigmatic example of an autoimmune disease that is characterized by the occurrence of ANA in virtually all patients, but for which interpretation of the patterns is complesx [17]. In fact, SSc ANA are mainly represented by four mutually exclusive specificities: anti-centromere (ACA), anti-topoisomerase I, anti-nucleolar, and anti-RNA polymerase III antibodies. Anti-PM-Scl, U1-RNP and anti-Ku are usually detected in overlap syndromes. About 60% of patients with SSc have ACA or anti-topoisomerase I antibodies as disease markers. Many other ANA that are present in SSc (for example, anti-RNA polymerase III, anti-Th/To, anti-PM/Scl, anti-Ku, anti-fibrillarin) are directed against different proteins localized in the nucleus and nucleolus. These antigen-antibody systems identify SSc subgroups with different evolution, organ involvement, and survival prognosis. The use of IIF for detection of ANA is mandatory for SSc diagnosis, displaying a sensitivity of 85% [1]. ACA and anti-topoisomerase I negative sera show strong anti-nuclear staining, featuring speckled or nucleolar (homogeneous, clumpy or speckled) patterns (Box 1). Therefore, the definition of the single nucleolar staining could address the suspect of specific autoantibodies, relevant for the diagnosis of SSc. A nucleolar ANA associated with new onset of Raynaud’s phenomenon could be helpful in identifying a patient with early disease, sometimes associated with severe organ involvement. It is essential that ANA results are confirmed by more specific methods such as western blotting or immunoprecipitation assays.

All these points underline the importance of correct interpretation of a given fluorescence pattern, and the need for standardization of analysis in automated systems.

There is one other important point about using automated systems for ANA reading. The ANA test was originally ordered predominantly by rheumatologists and clinical immunologists, but nowadays a broader range of clinical disciplines (including primary care, dermatology, nephrology, gastroenterology, neurology, oncology, hematology, obstetrics, gynecology, cardiology) are currently ordering the test. This change in test referral patterns affects the post-test probability for a given disease, as screening tests with limited specificity (such as IIF ANA) are strongly affected when the pre-test probability in a given population decreases [17]. A positive ANA test obtained outside of the rheumatologic setting displays poor predictive value for future development of a rheumatic disease, but it represents a significant risk factor for SLE. Taking into account that the prevalence of SLE is 1 in 2000 (0.05%), the observed frequency of 2.5% in individuals with a 1/80 positive ANA test represents a 50-fold relative risk for development of the disease [18,19]. Thus, ANA testing is a useful tool for SLE diagnosis.

## Conclusions

Current evidence from preliminary study results indicates that there is good correlation between manual and automated interpretation of ANA IIF assays, at least in the ability to discriminate between positive and negative results and in recognizing the main IIF patterns. Such systems will therefore speed up routine performance of these tests and help to harmonize interpretation of the results across laboratories. However, there is a need to have their clinical diagnostic power validated by clinical studies, in addition to the analytical studies that have already been published. In addition, these new systems could be further improved if they were better able to recognize mixed fluorescent or less common fluorescent patterns.

## Box 1 Anti-nuclear antibody assay

Best screening test for SLE

▪ Sensitivity ≥95%

▪ Specificity is only 57% for SLE compared with related rheumatic and autoimmune disorders

Key diagnostic assay for:

▪ SSc (sensitivity 85%)

▪ SS (sensitivity 48%)

▪ Drug-induced lupus (sensitivity 100%)

▪ PM/DM (sensitivity 61%)

▪ JIA (sensitivity 57%)

▪ MCTD (sensitivity 100%)

▪ Autoimmune hepatitis (sensitivity up to 60%)

Important role in assessing prognosis in Raynaud’s phenomenon [2].

DM, dermatomyositis; JIA, juvenile idiopathic arthritis; MCTD, mixed connective tissue disease; PM, polymyositis; SLE, systemic lupus erythematosus; SSc, systemic sclerosis; SS, Sjögren’s syndrome.

## Abbreviations

ACA: Anti-centromere antibodies; ANA: Anti-nuclear antibody; CAH: Chronic autoimmune hepatitis; CENP: Centromere protein; DFS70/LEDGF: Dense fine speckled/lens epithelium-derived growth factor; DM: Dermatomyositis; IIF: Indirect immunofluorescence; JIA: Juvenile idiopathic arthritis; MCTD: Mixed connective tissue disease; MSA: mitotic spindle apparatus; PAPS: Primary antiphospholipid syndrome; PBC: Primary biliary cirrhosis; PCNA: Proliferating cell nuclear antigen; PDH: Phosphate dehydrogenase; PM-Scl: Polymyositis-scleroderma; PM: Polymyositis; PNA: Positive/negative agreement; RA: Rheumatoid arthritis; SARDS: Systemic autoimmune rheumatic disease; SLE: Systemic lupus erythematosus; snRNP: Small nuclear ribonuclear protein; SSc: Systemic sclerosis; SS: Sjögren’s syndrome; UCTD: Undifferentiated connective tissue disease.

## Competing interests

PM has received fees as consultant for Inova and from BioRad; NB has been a paid consultant to Inova Diagnostics and has received lecture fees from A Menarini Diagnostics; and AT has received funding and reimbursements from IL, BioRad, and Thermofisher.

## Author contributions

All authors contributed equally to conception, design, acquisition of data, and analysis/interpretation of data; all have been involved in drafting and revising the manuscript; all have given final approval of the version to be published; and all agree to be accountable for all aspects of the work. All authors read and approved the final manuscript.

## Author information

PLM is Professor of Rheumatology at the University of Milan. MOB is deputy Director of the Experimental Immuno-Rheumatology Laboratory at the Istituto Auxologico Italiano. NB is Director of the Diagnostic Department at San Antonio Hospital, Tolmezzo. AT is Professor of Rheumatology at the University of Brescia and head of Rheumatology and Clinical Immunology Unit at Spedali Civili of Brescia. IC has a tenured position in Rheumatology and Clìnical lmmunology Unit at Spedali Civili of Brescia.
